# The Medical Society of the State of Ohio

**Published:** 1891-07

**Authors:** 


The Medical Society of the State of Ohio held its annual meeting for 1891 at Sandusky, June 17th, 18th, and 19th. The president, Dr. William J. Conklin, of Dayton, in his address, discoursed upon Moliere and the Doctors in a scholarly manner. It was a popular address to a popular audience, full of French classical research, and was received with hearty approbation, manifested in frequent applause. In an interesting discussion of the Surgical Treatment of Uterine Cancer, the hysterectomists seem to have vanquished their opponents in a handsome manner. The attendance upon the meeting was large, and the hospitality limitless.

